# MRI Radiomics in Imaging of Focal Hepatic Lesions: A Narrative Review

**DOI:** 10.7759/cureus.62570

**Published:** 2024-06-17

**Authors:** Nirupam Konwar Baishya, Kangkana Baishya, Kakoli Baishya, Rahul Sarma, Sushmita Ray

**Affiliations:** 1 Radiodiagnosis, Fakhruddin Ali Ahmed Medical College and Hospital, Barpeta, IND; 2 Electronics and Telecommunication, Assam Engineering College, Guwahati, IND; 3 Surgery, Guwahati Neurological Research Center (GNRC) Hospital, Guwahati, IND; 4 General Surgery, Fakhruddin Ali Ahmed Medical College and Hospital, Barpeta, IND

**Keywords:** artificial intelligence, imaging, focal hepatic lesions, radiomics, mri

## Abstract

Magnetic resonance imaging (MRI) is generally used to identify, describe, and evaluate treatment responses for focal hepatic lesions. However, the diagnosis and differentiation of such lesions require considerable input from radiologists. In order to reduce these difficulties, radiomics is an artificial intelligence (AI)-based quantitative method that employs the extraction of image features to reliably detect and differentiate focal hepatic lesions. MRI radiomics is a novel technique for the characterization of focal hepatic lesions. It can aid in preoperative evaluation, treatment approach, and forecast microvascular invasion. Although many studies have illustrated its efficiency there are certain limitations such as the absence of a large diverse dataset, comparison with other AI models, integration with histopathological findings, clinical utility, and feasibility.

## Introduction and background

Focal hepatic lesions represent a heterogeneous set of benign and malignant tumors with different pathogeneses, clinical manifestations, and prognoses. It is common practice to employ magnetic resonance imaging (MRI) to identify, describe, and evaluate treatment responses for these lesions [1]. However, due to aberrant presentations, uncommon tumor development over time, and overlap in imaging characteristics, characterizing such lesions can be difficult [1]. Subjective interpretation, inconsistent definitions, and reader experience all have an impact on the qualitative assessment of imaging features, which might result in less-than-ideal inter-reader agreement [1]. Differential diagnosis, predicting tumor aggressiveness, and assessing therapy response present difficulties for radiologists [1]. In recent years, various quantitative imaging methods have been proposed to overcome these difficulties. Radiomics is one such method that employs the extraction of image features to reliably detect and differentiate focal hepatic lesions.

## Review

Objective

The purpose of this article is to provide a comprehensive review of MR-based radiomics in the imaging of focal hepatic lesions.

Methods

An electronic search was carried out in various search engines like PubMed, Scopus, and Google Scholar, using keywords such as “radiomics,” “MRI,” “hepatic lesions,” “imaging,” “diagnosis," and their combinations. For this review, only articles published in English were considered. Nineteen articles were selected for detailed analysis based on relevance to the topic.

What is radiomics?

Radiomics is a novel radiological technique that permits decrypting medical imaging into minable numerical data and extracting high-throughput quantitative imaging features beyond human assessment [2]. Large datasets with radiomic features allow for the improvement of liver imaging assessment through sophisticated statistical models such as artificial intelligence (AI) and machine learning [1]. These models help explain the features of tumors, guide therapy choices, and enable prompt modifications to treatment plans [1]. Additionally, they tailor treatment plans to the specific needs of each patient [1].

Radiomics workflow

The radiomics workflow consists of several steps namely [3] - image acquisition, volumes of interest, segmentation, feature extraction, data integration and mining, and model building.

Image Acquisition

Relaxation times, scanner characteristics, acquisition settings, and image processing are variable parameters that influence the quality of images in MRI. Radiomics characteristics which depend on variations between voxel intensities instead of a particular voxel intensity would be ideal [4]. In order to mitigate this, a large data acquisition preferably from multiple centers and representing a diverse set of populations is beneficial.

Volumes of Interest

The identification of areas of interest within a tissue or a lesion is crucial for radiomic analysis. Different combinations of imaging data are used to form areas called “habitats” which have a particular set of parameters like the flow of blood, necrotic content, cellular density, etc. [3]. “Habitats” are actually subregions of the suspicious areas or lesions [3]. One of the main constraints in identifying such regions is the absence of a universal protocol for imaging [5]. Since the feasibility of such a protocol is daunting, the focus should be put on transparency of the protocols used [5].

Segmentation

There are mainly two types of segmentation which are used- semiautomatic and manual [6]. There are no proper guidelines as to which type should be used. Semiautomatic segmentations are generally robust but commonly images are segmented manually. Segmentation is a very important step in radiomics because the subsequent analysis is based on this particular step. Evaluation by different clinicians and application of different algorithms might prove to be useful in this regard [6].

Feature Extraction

Clinical images can be used to extract a variety of features, such as quantitative features extracted by software using mathematical algorithms, qualitative features describing the shape and voxel intensity histogram, and qualitative semantic features used in radiology to describe lesions [7]. Either straight from the photos or after using filters or other transformations, these features can be retrieved. Shape characteristics are a subset of quantitative features that characterize the geometric qualities and shape of the traced area of interest (ROI) [8]. Without taking spatial links into account, first-order statistics characteristics describe the distribution of individual voxel values [8]. Textural features, which are second-order statistical features, compute the statistical correlations between contiguous voxels and indicate the intra-lesion variability and spatial organization of voxel intensities [8]. After applying filters or mathematical transformations to the images, statistical techniques such as fractal analysis, Minkowski functionals, wavelet transform, and Laplacian transforms of Gaussian-filtered images yield characteristics of higher-order statistics [8]. Radiomics is a technique that quantifies image qualities by extracting many parameters from a single image. This approach, which was first created for fields studying molecular biology, processes the data using sophisticated statistical techniques. The use of textural features and filters in signal processing is the primary invention in radiomics [8]. The approach also leverages big-data analytics and extensive data-analysis expertise from other omics fields [8]. Choosing which and how many parameters to extract from photos presents challenges, though [9]. The amount of input variables determines the methods for data analysis, which may have an impact on the outcome [9]. A preliminary analysis to identify the most repeatable and reproducible parameters, reducing them through correlation and redundancy analysis, or making an a priori selection based on their mathematical definition are some methods [9]. Another is to start with all features offered by the calculation tool [9]. Machine learning methods are becoming used as practical instruments for feature selection.

Data Integration and Mining

Radiomics requires a large amount of high-quality data for training. Such datasets are not available easily. Most of the available datasets are retrospective and hence lack adequacy in the generalizability of results. There is a need for prospective datasets. The correlation between different radiomic features can be achieved through clustering.

Model Building

The three primary components of radiomic modeling are feature selection, modeling technique, and validation [10]. Incorporating information beyond radiomics, such as clinical records, treatment data, and biological/genetic data, feature selection should be robust and data-driven [10]. Selecting the right modeling methodology is essential, and a thorough radiomic study cannot be completed without validation [10]. Data-driven feature selection should be used to identify archetypal characteristics using dimensionality reduction approaches and remove features that are not resistant to variability [10]. Feature discretization is the process of converting continuous information into discrete binary interval features, and sample distribution correctness is measured through bootstrapping [10]. The modeling methodology that is selected frequently consists of a single, inherently limited technique [10]. While not necessary, using many modeling techniques is desirable. The modeling methodology that is selected frequently consists of a single, inherently limited technique [10]. The main measures used in validation approaches to evaluate the performance of the model are discrimination and calibration [10]. The best-case scenario would involve the use of several machine-learning techniques and thorough documentation of the implementation.

Radiomics quality score (RQS)

In order to evaluate previous and upcoming radiomic research and ensure adherence to best-practice protocols and justification for non-compliance, the RQS has been developed [11-13]. Publications should report research design, protocols, quality assurance procedures, and standard operating procedures in great detail, avoid making too optimistic promises about robustness and generalizability, and explicitly state how the study has progressed the profession by identifying unmet requirements [11-13]. For radiomics to advance, strict reporting criteria are required, and a growing number of publications now support and encourage the submission of substantial supplemental materials [11-13]. In order to reduce bias and improve the utility of prediction models, the RQS also addresses the inadequate reporting of prediction model research, mandating complete and unambiguous reporting of data [11-13]. A case in point is the endeavor known as Transparent Reporting of a Multi-Variable Prediction Model for Individual Prognosis or Diagnosis (TRIPOD) [14].

MRI radiomics in differentiation between the benign and malignant type of focal hepatic lesions

Starmans et al. created a radiomics model to use (MRI) to differentiate between benign and malignant primary solid liver lesions [15]. Between 2002 and 2018, 486 patients from three tertiary referral hospitals were used to evaluate the machine learning-developed model [15]. The findings revealed an internal validation mean area under the curve (AUC) of 0.78 and an external validation AUC of 0.74 and 0.76 [15]. In addition, it was discovered that the model could be used for a wide range of MRI acquisition procedures [15]. The research indicates that radiologists may be able to enhance the diagnostic process for patients with liver abnormalities by pursuing additional optimization and generalization. There is substantial potential for the model to discriminate between benign and malignant lesions [15]. The purpose of the study by Wu et al. was to assess the classification of hepatic hemangioma (HH) and hepatocellular carcinoma (HCC) using radiomics and precontrast MRI [16]. From magnetic resonance scans of HCC and HH, the researchers retrieved 1,029 radiomics characteristics after enrolling 369 consecutive patients with 446 lesions [16]. Radiomics characteristics were utilized to identify HH and HCC using four classifiers [16]. Additionally, two abdominal radiologists conducted a qualitative categorization analysis [16]. Receiver operating characteristic (ROC) analysis was used to assess the diagnostic performances [16]. The findings demonstrated that when four sequences were added, the logistic regression classifier had superior predictive power [16]. There was no statistical difference in the diagnostic performance of the optimal radiomics-based combined model between experienced radiologists (10-year experience) and less experienced radiologists (2-year experience) [16]. According to the study's findings, a radiomics signature can be created and verified as an additional tool for differentiating between HH and HCC [16]. In order to differentiate hepatic epithelioid angiomyolipoma (HEAML) from HCC and focal nodular hyperplasia (FNH), two radiomics-based models were developed and validated by Liang et al. [17]. Preoperative contrast-enhanced CT and MRI scan data from 170 and 137 patients, respectively, were used in the study [17]. From each patient's ROI, quantitative texture and wavelet features were recovered [17]. The random forest approach was utilized to create radiomic signatures, while multivariate linear regression and 10-fold cross-validation were employed to develop fusion models [17]. The outcomes demonstrated that the random forest algorithm-based radiomics signatures had the best prediction performance in both CT and MRI data [17]. It was discovered that the fusion models were very good at differentiating HEAML from FNH and HCC, which suggests that they could be used as diagnostic tools for customized treatment plans [17]. The purpose of the study by Zhao et al. was to create and verify a radiomics-based model for the preoperative distinction between HCC and fat-poor angiomyolipoma (fp-AML) in noncirrhotic livers using contrast-enhanced MRI (CE-MRI) [18]. A dataset including three cohorts - a training cohort, an internal validation cohort, and an external validation cohort - was used to evaluate the model on 165 patients from three different medical centers [18]. The combined model outperformed the other models in terms of AUC [18]. The combined model's diagnostic accuracy, sensitivity, and specificity were better than the two radiologists' respective ones [18]. In both validation cohorts, the combined model outperformed the models of the two radiologists and was much higher than the junior model [18]. The objective of the work by Zhang et al. was to assess the variability of radiomic characteristics in HCC that were retrieved from several diffusion-weighted images (DWIs) with b-values [19]. Twelve healthy volunteers and 34 HCC patients made up the research population [19]. Sequences were performed on each case at 10 b-values between 0 and 1,500 s/mm2 [19]. For every case series, 74 radiomic characteristics 19 first-order statistical features, and 55 texture features were retrieved using the 3D Slicer Radiomics software [19]. The research discovered that b-values in HCC were a determining factor in the intensity histogram characteristics and texture features obtained from DWIs [19]. About 26%, 28%, and 46% of the radiomic properties were accounted for by low variations (%COV <30), moderate changes (30≤ %COV <50), and significant variations (%COV ≥50) [19]. A minor dependence was seen in about 4% of radiomic characteristics, but about 70% exhibited positive or negative dependence on b-values [19]. Additionally, the study discovered that radiomic characteristics with good repeatability were retrieved from DWIs' neighboring b-values [19]. Hepatic cancer and normal liver can be distinguished using 12 radiomic characteristics [19]. The study by Oyama et al. assesses the precision of T1-weighted MRI in the categorization of liver cancers [20]. Fifty HCCs, 50 metastatic tumors (MTs), and 50 HHs were among the 150 hepatic tumors evaluated in the study. For categorization, persistence pictures and texture features were computed [20]. There were three models of classification in use [20]. The study discovered that employing texture analysis, HCC, and MT categorization had an accuracy of 92%, with degree 1 persistence images yielding the highest accuracy of 85% [20]. These techniques enable the classification of hepatic tumors with a high degree of accuracy, which may be helpful for computer-aided diagnosis using MRI [20]. The purpose of the research by Jansen MJA, Kuijf HJ, Veldhuis WB, Wessels FJ, Viergever MA, Pluim JPW was to evaluate the utility of gadoxetate disodium-enhanced MRI in the differential diagnosis of hepatic cavernous hemangioma (HHE) and HCC [21]. Radiomic texture parameters were evaluated for classification and differentiation using data gathered from 135 HCC and HHE lesions [21]. Over 50% of radiomic features had substantial differentiating power, according to the results, with the gray-level co-occurrence matrix feature SumEntrp doing well in classification [21]. For multivariate analysis, the SFS algorithm was used since it produced superior results to alternative algorithms [21]. The study suggests that HCC and HHE can be effectively diagnosed using gadoxetate disodium-enhanced MRI radiomic characteristics, which may help with clinical diagnosis [21].

MRI radiomics in the prediction of microvascular invasion (MVI)

The goal of the study by Feng et al. was to create and validate a combined intratumoral and peritumoral radiomics model for primary HCC patients that used gadolinium-ethoxybenzyl-diethylenetriamine (Gd-EOB-DTPA) enhanced MRI to predict MVI prior to surgery [22]. One hundred ten HCC patients and 50 HCC patients participated in the study [22]. They had a curative hepatectomy and a preoperative Gd-EOB-DTPA enhanced MRI evaluation. 38.2% and 40.0% of the patients were MVI-positive, respectively [22]. Ten features were chosen by supervised machine learning to create an MVI prediction model [22]. With its excellent AUC, sensitivity, and specificity, combined intratumoral and peritumoral radionics model assisted doctors in making accurate treatment decisions prior to surgery [22]. In a study by Chong et al., MVI and recurrence-free survival (RFS) were predicted using radiomics-based nomograms for 356 patients with solitary HCC < 5 cm [23]. In the validation cohort, the AUCs of the MVI nomogram were 0.879 by logistic regression analysis and 0.920 by random forest [23]. Patients with an MVI had a median RFS of 30.5 months and > 96.9 months, respectively [23]. Recurrence was independently predicted by age, histologic MVI, alkaline phosphatase, and alanine aminotransferase, with an AUC of 0.654 in the RFS validation sample [23]. According to the study's findings, preoperative radiomics-based nomograms that use random forests could be used as biomarkers to predict MVI and RFS [23].

MRI radiomics in hepatic metastasis

The objective of the study by Xu et al. was to develop a radiomics nomogram that would distinguish intrahepatic mass-forming cholangiocarcinoma (IMCC) from colorectal cancer liver metastasis (CRLM) using multiparameter MRI [24]. A training cohort of 133 patients, an internal validation cohort of 57 patients, and an external validation cohort of 51 patients were all included in the study [24]. The least absolute shrinkage and selection operator algorithm was utilized to extract and select radiomic features from the images [24]. A clinical model was built using MRI results and clinical factors [24]. The radiomics model was built using six characteristics, and in both the training and external validation cohorts, the radiomics signature outperformed the clinical model in terms of discriminating [24]. Preoperatively, the radiomics nomogram may prove to be a dependable and noninvasive means of prognosticating treatment plans [24]. The purpose of the study by Granata et al. was to determine how well radiomics features from portal and arterial MRI phases may predict clinical outcomes for patients with colorectal liver metastases [25]. Retrospective investigation of individuals with pathologically proven colorectal liver metastases and MRI examination in a preoperative context following neoadjuvant treatment were part of the study [25]. The cohort comprised 30 patients with a single lesion and a median age of 60 years, as well as 51 patients with 121 liver metastases and a median age of 61 years [25]. The study came to the conclusion that radiomics can provide a more individualized approach by identifying biomarkers and prognostic factors that may influence treatment decisions in individuals with liver metastases [25].

By obtaining radiomic characteristics and pharmacokinetic information, the study by Li et al. sought to determine whether HCC and hepatic metastasis of rectal cancer (HMRC) could be distinguished using dynamic contrast-enhanced MRI (DCE-MRI) [26]. DCE-MRI was performed on 75 patients, of whom 34 were HMRC cases and 41 were HCC cases [26]. Pharmacokinetic parameters and radiomic characteristics were computed using a dual-input, tracer kinetic model and specialized picture post-processing software in this investigation [26]. The hepatic perfusion index, endothelium transfer constant, and initial area under the gadolinium concentration curve (IAUC) within the first 60 seconds (IAUC) between the HCC and HMRC groups were found to differ significantly [26]. In addition, there were statistically significant variations between the two groups in 17 radiomic characteristics [26]. Based on radiomic characteristics, Fisher's discriminant analysis model has an accuracy of 89.3% [26]. The purpose of the study by Shu et al. was to use radiomics to predict synchronous liver metastasis (SLM) in primary rectal cancer [27]. One hundred ninety-four patients' T2WI scans yielded 328 radiomics characteristics [27]. To minimize the feature dimension and create the radiomics signature, the least absolute shrinkage and selection operator (LASSO) regression method was employed [27]. To choose features with an 85% contribution, surplus characteristics were sorted using principal component analysis (PCA) [27]. The net benefit was assessed using decision curve analysis and a linear regression prediction model [27]. The model with the highest net benefit was the one based on the LASSO dimensionality building [27]. When paired with LASSO characteristics and clinical risk factors, the radiomics nomogram demonstrated strong predictive ability [27]. According to this, radiomics based on primary rectal cancer may offer a non-invasive method of estimating the risk of SLM in clinical settings [27]. In order to assess the HMRC, the study by Hu et al. sought to determine the clinical utility of employing radiomics models based on various MRI sequences [28]. Baseline MRI was performed on 140 individuals with pathologically diagnosed rectal cancer between April 2015 and May 2018 [28]. Based on the findings of the imaging tests, surgical pathology, and liver biopsy, the patients were split into two groups [28]. Using the software, logistic regression models including specific radiomic features were created for the training and test cohorts of data [28]. The study found that hepatic metastasis may be accurately assessed by the combined model (T2WI+DWI+ADC), the T2WI model, and the ADC model; the train set's AUC was 93.5%, 89.2%, and 90.6%, while the test set's AUC was 80.8%, 80.5%, and 81.4%, respectively [28]. With a high AUC that was on par with the T2WI and ADC models and the best fit to the calibration curve's diagonal reference line, the combined model performed the best [28]. The purpose of the study by Shahveranova et al. was to use integrated models based on clinical features and MRI radiomics to predict local tumor progression (LTP) in patients with CRLM [29]. With regard to the 67 tumors in 42 consecutive CRLM patients, the study collected 11 radiomics features for every phase and tumor [29]. Two integrated models were made using feature reduction and machine learning, and a clinical model was built using clinical data [29]. According to the findings, 16.6% of patients and 16.4% of tumors had LTP [29]. Before microwave ablation, the existence of extrahepatic metastases was linked to a higher risk of LTP [29]. In both periods, radiomics scores were considerably higher in patients with LTP [29]. In terms of predicting LTP, the combination model that included clinical data and Phase 2-based radiomics features had the best discriminative performance [29]. 

Radiomic-clinical integration

The purpose of the study by Liu et al. was to determine how well MRI radiomics can differentiate intrahepatic cholangiocarcinoma (ICC) from HCC [30]. One hundred twenty-nine HCC patients and 48 ICC patients provided data for analysis. Radiomics characteristics were extracted from axial fat suppression T2-weighted imaging (FS-T2WI), axial arterial-phase (AP), and portal-venous-phase (PVP) DCE-MRI sequences using a 7:3 training and validation group [30]. The optimal radiomic characteristics were chosen using the LASSO approach [30]. With every sequence, radiomic models were created using logistic regression [30]. These models included a joint radiomics model (JR model), a clinical model with optimal clinical variables (C model), and a radiomics-clinical model incorporating ideal radiomic characteristics [30]. According to the findings, MRI-based radiomics may be able to assist in the noninvasive separation of ICC and HCC [30]. The goal of the study by Yang et al. was to create a radiomics nomogram based on T2-weighted imaging that could distinguish between benign liver lesions with rich blood supply and hepatocellular cancer [31]. One hundred forty-four individuals with benign blood-rich liver lesions and hepatocellular cancer had their imaging and clinical data examined [31]. Three prediction models were created: a fusion model that integrated clinical and radiomic parameters, a clinical model, and a radiomic model [31]. The most accurate prediction model was compared to junior and senior radiologists' diagnostic performance [31]. In both the training and validation sets, the fusion model significantly outperformed senior and younger radiologists in terms of discrimination capabilities [31]. By avoiding the need for repeating contrast chemicals and enhancing conventional imaging diagnosis, the T2WI-based radiomics nomogram enables early clinical diagnosis and targeted treatment [31].

Comparison of MRI radiomics models with Liver Imaging Reporting and Data System (LI-RADS) and European Association for the Study of the Liver (EASL) diagnostic criteria

Jiang et al. assessed the diagnostic accuracy of radiomics models, the EASL criteria, and the LI-RADS for HCC in high-risk patients [32]. Two hundred eleven individuals had liver surgery after receiving gadoxetic acid-enhanced MRI between July 2015 and September 2018 [32]. A three-dimensional, multi-sequence whole-tumor radiomics signature was created and verified in a separate group of patients [32]. The outcomes demonstrated 86 and 82% for the LI-RADS criteria, 91 and 71% for the EASL criteria, and 73 and 71% for the radiomics signature, respectively [32]. Comparable AUCs were found for the LI-RADS, EASL, and radiomics signature criteria [32].

Discussion

Radiomics might be used to identify liver metastases, particularly hepatic malignancy. The distinction between benign and malignant tumors can be made and confirmed using a radiomics signature. Prior to surgery, the combined intratumoral and peritumoral radionics model can help physicians make well-informed treatment decisions. Hepatic tumors, including liver metastases, may be reliably distinguished from non-hepatic tumors (HCC) and liver carcinoma (HHE) using fusion models. Nomograms based on preoperative radiomics can be utilized as biomarkers to forecast RFS and MVI. Additionally, by identifying biomarkers and prognostic factors that affect treatment decisions, radiomics can offer a more customized approach. Noninvasive ICC and HCC separation may be aided by MRI-based radiomics while HCC and HHE can be successfully diagnosed using gadoxetate disodium-enhanced MRI radiomic features, which may help with clinical diagnosis. Delta radiomics texture features can detect and quantify the underlying biological changes brought on by radiation delivery in hepatic lesions.

Although many studies have illustrated its efficiency, there are certain limitations like the absence of a large dataset, demographic variability in patients, multicentric data requirement, comparison with other AI models like deep learning convolutional neural networks, integration with histopathological findings, outcome precision, overfitting or underfitting, clinical utility, feasibility and studies involving different types of hepatic lesions.

## Conclusions

MRI radiomics is a novel technique for characterizing focal hepatic lesions. It can aid in preoperative evaluation, treatment approach, and forecast microvascular invasion. The distinction and characterization of benign, as well as malignant focal hepatic lesions with integration into clinical practice, is the ultimate aim of radiomics. However, to do so, it is necessary to conduct further research that will address important limitations in this field such as the presence of a diverse dataset that is generalizable, minimal or absent overfitting or underfitting problem, and clinical feasibility.
